# Long-term risk of incident dementia following documented COVID-19 among older adults with type 2 diabetes: a propensity score-matched cohort study

**DOI:** 10.3389/fnagi.2026.1927383

**Published:** 2026-09-15

**Authors:** I-Wen Chen, Li-Chen Chang, Chun-Ning Ho, Kuo-Chuan Hung

**Affiliations:** 1 Department of Anesthesiology, Chi Mei Hospital, Liouying, Tainan City, Taiwan; 2 Department of Anesthesiology, E-Da Hospital, I-Shou University, Kaohsiung City, Taiwan; 3 Department of Anesthesiology, Chi Mei Medical Center, Tainan City, Taiwan; 4 School of Medicine, College of Medicine, National Sun Yat-sen University, Kaohsiung City, Taiwan

**Keywords:** cohort study, COVID-19, dementia, propensity score matching, TriNetX, type 2 diabetes mellitus

## Abstract

**Background:**

Type 2 diabetes mellitus (T2DM) is a well-established risk factor for dementia, and COVID-19 has been associated with persistent neurological and cognitive sequelae. However, whether this association extends beyond the early post-infection period among older adults with T2DM—a population with compounded vascular and metabolic vulnerability—remains unclear.

**Methods:**

Using the TriNetX Global Collaborative Network, we conducted a retrospective cohort study of patients aged ≥65 years with T2DM, comparing those with documented COVID-19 to a matched unexposed cohort (1:1 propensity score matching). A 1-year landmark design was applied, with follow-up extending to 6 years after the index date. The primary outcome was incident overall dementia, which is a composite of vascular dementia, Alzheimer’s disease, and unspecified/other dementia. The secondary outcomes included individual dementia subtypes, mild cognitive impairment, all-cause mortality, and cerebral infarction.

**Results:**

After matching, 449,745 patients were included in each group with well-balanced covariates (all standardized mean differences <0.1). Documented COVID-19 was associated with a significantly increased risk of overall dementia [hazard ratio (HR 1.51), 95% confidence interval (CI) 1.47–1.55, *p* < 0.001]. The secondary outcomes were similarly elevated, such as vascular dementia (HR 1.61), Alzheimer’s disease (HR 1.41), mild cognitive impairment (HR 1.66), all-cause mortality (HR 1.56), and cerebral infarction (HR 1.33; all *p* < 0.001). The hazard ratio for overall dementia increased progressively across sequential landmark periods (1.51 at 1 year, 1.66 at 2 years, and 2.09 at 3 years), and the findings remained robust across sensitivity analyses, addressing survival, healthcare utilization, and exposure misclassification. The findings were consistent in separately constructed and propensity score-matched additional cohorts restricted to adults aged 50–65 years and to patients indexed during the Omicron-predominant period.

**Conclusion:**

In this large propensity score-matched cohort study, documented COVID-19 was associated with an increased long-term risk of incident dementia among older adults with T2DM, with the association persisting and strengthening beyond the early post-infection period. These findings support sustained attention to cognitive health in this metabolically susceptible population following COVID-19; however prospective studies incorporating standardized cognitive and biomarker assessments are needed to clarify underlying causes.

## 1 Introduction

Dementia is an escalating global health burden, driven largely by population aging, with recent projections indicating sustained increases in Alzheimer’s disease and other dementias through 2040 and rising early-onset dementia cases and dementia-related economic burden through 2050 (Feng et al., 2025; Hao and Chen, 2025; Nandi et al., 2022; Twiss et al., 2025). Type 2 diabetes mellitus (T2DM) is a well-established risk factor for dementia, with the association reflecting multiple interrelated pathways and clinical factors, including chronic hyperglycemia, glycemic control, hypoglycemia, diabetic microvascular and macrovascular complications, cerebral microvascular injury, accelerated neurodegeneration, and antidiabetic medication use (Bello-Chavolla et al., 2019; Singh et al., 2022; Cherbuin and Walsh, 2019; Mohamed-Mohamed et al., 2023; Tseng, 2026). Age at diabetes onset may also influence dementia development, with earlier-onset diabetes associated with a higher subsequent risk (Tseng, 2026). Older adults with T2DM, therefore, represent a high-priority population for evaluating post-COVID dementia risk because advanced age and T2DM impose overlapping metabolic, neuroinflammatory, and cerebrovascular vulnerabilities. In this already high-risk population, any additional damage to cerebrovascular or neuroinflammatory pathways could result in a measurable increase in dementia incidence. Beyond acute respiratory illness, COVID-19 has been associated with persistent multisystem sequelae, such as neurological and cognitive complications, collectively recognized as post-acute sequelae of SARS-CoV-2 infection (Parotto et al., 2023; Moghimi et al., 2021; Ong et al., 2023; Walitt and Johnson, 2022). Emerging evidence has suggested that SARS-CoV-2 infection can trigger sustained neuroinflammation, endothelial dysfunction, and blood–brain barrier disruption (Reynolds and Mahajan, 2021; Alquisiras-Burgos et al., 2021; Cárdenas et al., 2022), mechanisms that overlap substantially with those implicated in diabetes-related cognitive decline.

Recent systematic reviews and meta-analyses (Zhang et al., 2025; Shan et al., 2024; Shrestha et al., 2024) have reported an increased risk of incident dementia or cognitive impairment following COVID-19 in older or mixed adult populations; however, whether this association persists among older adults with pre-existing T2DM, a population with compounded vascular and metabolic vulnerability, remains poorly characterized. Moreover, nearly all existing evidence has been limited to follow-up windows of 6 months to 2 years (Zhang et al., 2025; Shan et al., 2024; Shrestha et al., 2024), leaving unresolved whether the dementia risk observed after COVID-19 reflects a true, latency-dependent process that continues beyond this window or largely reflects short-term detection bias from increased post-COVID medical surveillance. Detection bias is particularly plausible in this context since COVID-19 survivors often have more frequent healthcare contact during and after recovery, increasing the likelihood that pre-existing or evolving cognitive impairment is incidentally diagnosed.

This evidence gap is increasingly important as population aging expands the number of older adults living with both T2DM and prior COVID-19 exposure. To date, few studies have provided long-term follow-up beyond the early post-infection period, and the majority of them have not incorporated analytic strategies specifically designed to address detection bias, early outcome ascertainment, or competing mortality. In older adults with T2DM, these methodological concerns are especially relevant because this population has high baseline dementia risk, frequent healthcare utilization, substantial vascular vulnerability, and elevated competing mortality. Therefore, distinguishing a persistent post-COVID dementia association from a surveillance-related artifact requires longer follow-up and complementary bias-mitigation approaches. To address this gap, we conducted a propensity score-matched cohort study with the primary objective of determining whether documented COVID-19 was associated with an increased long-term risk of incident overall dementia after a 1-year landmark period among adults aged ≥65 years with T2DM. Follow-up extended to 6 years after the index date, with sequential landmark analyses, negative-control outcomes, and a competing-risk framework incorporated to address potential biases.

## 2 Methods

### 2.1 Study design and data source

This retrospective cohort study used the TriNetX Global Collaborative Network, a federated electronic health record platform aggregating de-identified data from 181 participating healthcare organizations worldwide. This database has been widely used in numerous published studies across diverse clinical fields (Hung et al., 2026; Chen et al., 2026; Chen et al., 2025). The study protocol was approved by the Institutional Review Board of Chi Mei Medical Center, and the requirement for informed consent was waived. The study was conducted in accordance with the Declaration of Helsinki.

### 2.2 Study population and exposure definition

Eligible patients were aged ≥65 years with a documented diagnosis of type 2 diabetes mellitus (T2DM; ICD-10-CM E11) recorded between 1 January 2020 and 30 June 2022. Within this population, the exposed cohort comprised patients who additionally received a diagnosis of COVID-19 (ICD-10-CM U07.1) or a positive SARS-CoV-2 RNA test during the same period; the index date was the first date on which both criteria were satisfied. The unexposed cohort included T2DM patients whose index date was defined as the date of the first qualifying T2DM diagnosis recorded between 1 January 2020 and 30 June 2022, with no concurrent COVID-19 diagnostic or positive SARS-CoV-2 RNA test documented. A 1-year landmark design was applied after the index date to reduce detection-related bias arising from dementia diagnosed shortly after COVID-19 was confirmed. Patients diagnosed with dementia before or during the first 365 days after the index date and those who died during the same period were excluded from the study, so only patients who were alive and dementia-free at day 365 entered the analytic risk set. Follow-up began at this landmark and continued until dementia diagnosis, death, the last available record, or 6 years after the index date, whichever occurred first.

### 2.3 Exclusion criteria

Patients with dementia (vascular dementia, F01; dementia in other diseases classified elsewhere, F02; unspecified dementia, F03; or other degenerative diseases of the nervous system, G30–G32) recorded before or within 1 year after the index date were excluded to eliminate prevalent or immediately antecedent dementia. Patients who died or had an ill-defined/unknown cause of death (R99) within 1 year after the index date were excluded, consistent with the landmark’s minimum 1-year survival requirement. To ensure diagnostic homogeneity of the T2DM population, patients with type 1 diabetes were also excluded from the study. To further reduce confounding from conditions that predispose to, clinically overlap with, or are pharmacologically managed similarly to dementia, patients with pre-index chronic kidney disease stage 4–5/dialysis dependence, intracranial injury, multiple sclerosis, epilepsy, schizophrenia, bipolar disorder, Parkinson’s disease, prior use of cholinesterase inhibitors or memantine, or hearing loss were also excluded. Codes used to define exclusion criteria are shown in Supplementary Table 1.

### 2.4 Propensity score matching

Cohorts were matched 1:1 using a greedy nearest-neighbor algorithm with a caliper of 0.1 pooled standard deviations of the propensity score logit. The matching model incorporated covariates selected *a priori* to balance established or plausible dementia risk factors and factors associated with COVID-19 susceptibility, severity, healthcare utilization, or outcome ascertainment, such as demographics (age, sex, and race); obesity and a broad panel of cardiovascular, renal, hepatic, respiratory, psychiatric, and metabolic comorbidities, such as diabetes-related renal, neurological, circulatory, and ophthalmic complications, anemia, malnutrition, and frailty indicators (repeated falls and care dependency); concomitant medications, such as antilipemic agents, insulins, metformin, sodium-glucose cotransporter 2 (SGLT2) inhibitors, glucagon-like peptide-1 (GLP-1) receptor agonists, glucocorticoids, and COVID-19 vaccination; baseline laboratory values such as BMI, albumin, eGFR, hemoglobin, and HbA1c; and healthcare utilization such as emergency visits, hospitalization, and critical care. Covariate balance was assessed using standardized mean differences (SMDs), with values <0.10 considered acceptable. Complete definitions are provided in Supplementary Table 2.

### 2.5 Outcomes

The primary outcome was incident overall dementia, which is a composite of vascular dementia (ICD-10-CM F01), dementia in other diseases classified elsewhere (F02), unspecified dementia (F03), and Alzheimer’s disease (G30). The secondary outcomes included the individual dementia subtypes comprising the primary composite, such as vascular dementia and Alzheimer’s disease, together with mild cognitive impairment, as well as all-cause mortality and cerebral infarction/stroke.

The outcomes were ascertained in the window beginning 365 days and ending 2,190 days (6 years) after the index date. To assess detection-related bias, we evaluated two negative-control outcomes—age-related cataract and benign skin neoplasm. Persistent between-group differences in these outcomes would suggest that the observed findings may reflect differential healthcare-seeking or diagnostic intensity rather than a specific association between COVID-19 and dementia. The overall healthcare visit frequency was compared as an indicator of surveillance intensity.

### 2.6 Sensitivity and subgroup analyses

We conducted three sensitivity analyses to evaluate the robustness of the findings against survival-related, surveillance-related, and exposure-misclassification artifacts. Models I and II were applied symmetrically to both cohorts. Model I included only patients who survived throughout follow-up, whereas Model II further restricted the analysis to patients with at least one healthcare visit during follow-up, thereby addressing differential mortality and healthcare-seeking as potential sources of bias. Model III addressed potential contamination of the unexposed cohort by restricting controls to individuals without documented COVID-19 during follow-up, as crossover from the control group to COVID-19 exposure would be expected to attenuate the estimated association toward the null.

Prespecified subgroup analyses were stratified by sex, vaccination status, and presence/absence of hypertension, chronic kidney disease, obesity, dyslipidemia, cerebrovascular disease, sleep disorders, nicotine dependence, and glycemic control (HbA1c ≥ 9% vs. <9%), with interaction terms tested.

### 2.7 Landmark sweep and competing-risk analyses

To determine whether the association reflected a latency-dependent biological effect rather than short-term surveillance bias, a landmark sweep was performed using 2- and 3-year event-free landmark points, excluding patients who experienced the outcome or death during each landmark period. As the TriNetX platform does not support a formal competing-risk regression analysis with between-group statistical testing (e.g., Fine–Gray sub-distribution hazard models), a descriptive competing-risk analysis was additionally performed in the unmatched cohort using the Aalen–Johansen estimator, with death treated as a competing event, without formal between-group comparison.

### 2.8 Additional analysis

To further evaluate the generalizability and robustness of the primary findings, two additional cohorts were separately assembled and propensity score-matched independently of the primary cohort aged ≥65 years. Both analyses used the same exposure and outcome definitions, covariate set, matching algorithm, and caliper as described above. First, a younger cohort aged 50–65 years was separately constructed and propensity score-matched to assess whether the observed association was specific to older adults, in whom greater healthcare surveillance and comorbidity assessment may increase the potential for detection bias. Second, a separate cohort was assembled using a later index period from 1 July 2022 to 30 June 2023, corresponding to the predominance of Omicron-lineage variants. This analysis was performed to examine whether the association was consistent across different phases of the pandemic and whether the findings observed during earlier, more virulent variant periods extended to the Omicron era.

### 2.9 Statistical analysis

Baseline characteristics were summarized as means ± standard deviations for continuous variables and frequencies (%) for categorical variables. In matched cohorts, time-to-event associations were estimated using Cox proportional hazards models, with hazard ratios (HRs) and 95% confidence intervals (CIs) reported alongside log-rank tests; proportional hazards assumptions were evaluated using the TriNetX platform’s built-in proportionality test. For the primary outcome, E-values were calculated to quantify the minimum strength of association an unmeasured confounder would need with both exposure and outcome to fully explain the observed estimate and its lower confidence bound. Missing data were not imputed. A two-sided *p*-value of less than 0.05 was considered significant for the primary outcome; secondary, sensitivity, subgroup, landmark, and competing-risk analyses were regarded as exploratory, and no adjustment for multiple comparisons was applied. All analyses were performed within the TriNetX platform (TriNetX LLC, Cambridge, MA).

## 3 Results

### 3.1 Cohort assembly and covariate balance

Among patients aged ≥65 years with type 2 diabetes identified in the TriNetX network, 523,910 patients with documented COVID-19 and 955,316 unexposed patients met eligibility criteria before matching (Table 1). After 1:1 propensity score matching, 449,745 patients remained in each group. Before matching, the COVID-19 group differed from controls across multiple comorbidities, medications, and laboratory parameters (SMD up to 0.524 for hemoglobin ≥12 g/dL). After matching, covariate balance was excellent, with all SMDs <0.1, indicating adequate control of measured confounding. After propensity score matching, the mean follow-up duration was 3.61 ± 1.77 years in the COVID-19 group and 4.13 ± 1.86 years in the control group. The median follow-up duration was 4.12 years in the COVID-19 group, and it was 4.60 years in the control group.

**Table 1:** Baseline characteristics of older adults with type 2 diabetes with and without documented COVID-19.

Variables	Before matching			After matching		
	COVID-19 group (*n* = 523,910)	Control group (*n* = 955,316)	SMD	COVID-19 group (*n* = 449,745)	Control group (*n* = 449,745)	SMD
Patient characteristics						
Age at index (years)	70.6 ± 7.6	70.2 ± 7.6	0.050	70.5 ± 7.6	70.4 ± 7.6	0.009
Female	259,844 (49.6)	478,713 (50.1)	0.010	223,146 (49.6)	222,152 (49.4)	0.004
BMI ≥ 30 kg/m^2^	242,080 (46.2)	291,908 (30.6)	0.326	191,901 (42.7)	197,614 (43.9)	0.026
White	331,568 (63.3)	505,780 (52.9)	0.211	276,359 (61.5)	282,187 (62.7)	0.027
Black or African American	84,171 (16.1)	127,197 (13.3)	0.078	70,452 (15.7)	72,604 (16.1)	0.013
Asian	25,740 (4.9)	62,688 (6.6)	0.071	23,979 (5.3)	26,100 (5.8)	0.021
Comorbidities						
Hypertension	361,422 (69.0)	461,058 (48.3)	0.430	292,737 (65.1)	298,374 (66.3)	0.026
Dyslipidemia	319,218 (60.9)	412,489 (43.2)	0.361	256,436 (57.0)	260,248 (57.9)	0.017
Neoplasms	163,963 (31.3)	187,742 (19.7)	0.270	126,284 (28.1)	128,549 (28.6)	0.011
Overweight and obesity	151,221 (28.9)	151,487 (15.9)	0.316	111,946 (24.9)	111,972 (24.9)	0.000
Ischemic heart diseases	146,753 (28.0)	151,283 (15.8)	0.298	107,710 (24.0)	107,265 (23.9)	0.002
Sleep disorders	115,293 (22.0)	110,004 (11.5)	0.284	82,180 (18.3)	81,191 (18.1)	0.006
Chronic pain	93,301 (17.8)	79,867 (8.4)	0.283	64,087 (14.3)	63,191 (14.1)	0.006
Vitamin D deficiency	73,555 (14.0)	89,380 (9.4)	0.146	57,608 (12.8)	58,223 (13.0)	0.004
Chronic kidney disease (CKD)	80,285 (15.3)	78,472 (8.2)	0.222	57,367 (12.8)	56,803 (12.6)	0.004
Anxiety disorders	72,860 (13.9)	67,959 (7.1)	0.223	51,074 (11.4)	50,202 (11.2)	0.006
T2DM with kidney complications	65,945 (12.6)	66,275 (6.9)	0.191	46,951 (10.4)	46,540 (10.4)	0.003
Anemias	70,263 (13.4)	58,340 (6.1)	0.248	46,947 (10.4)	46,021 (10.2)	0.007
Heart failure	70,751 (13.5)	52,007 (5.4)	0.278	44,736 (10.0)	42,989 (9.6)	0.013
T2DM with neurological complications	62,028 (11.8)	59,061 (6.2)	0.199	43,610 (9.7)	43,321 (9.6)	0.002
Cerebrovascular diseases	58,849 (11.2)	59,818 (6.3)	0.177	42,555 (9.5)	42,275 (9.4)	0.002
Diseases of the liver	55,712 (10.6)	56,816 (6.0)	0.171	40,381 (9.0)	39,985 (8.9)	0.003
Other chronic obstructive pulmonary disease	61,598 (11.8)	47,754 (5.0)	0.246	39,579 (8.8)	38,453 (8.6)	0.009
Nicotine dependence	54,757 (10.5)	50,522 (5.3)	0.193	38,807 (8.6)	38,378 (8.5)	0.003
T2DM with circulatory complications	33,767 (6.5)	29,073 (3.0)	0.161	22,858 (5.1)	22,460 (5.0)	0.004
T2DM with ophthalmic complications	27,083 (5.2)	33,657 (3.5)	0.081	20,621 (4.6)	20,924 (4.7)	0.003
Sepsis	22,075 (4.2)	13,491 (1.4)	0.170	12,755 (2.8)	11,837 (2.6)	0.013
Major depressive disorder	17,177 (3.3)	14,598 (1.5)	0.114	11,590 (2.6)	11,331 (2.5)	0.004
Alcohol-related disorders	12,546 (2.4)	9,386 (1.0)	0.110	8,045 (1.8)	7,730 (1.7)	0.005
Problems related to care provider dependency	9,487 (1.8)	7,118 (0.8)	0.095	6,108 (1.4)	5,897 (1.3)	0.004
Repeated falls	8,599 (1.6)	5,193 (0.5)	0.106	4,705 (1.1)	4,541 (1.0)	0.004
Malnutrition	8,621 (1.7)	4,178 (0.4)	0.119	4,370 (1.0)	3,879 (0.9)	0.011
Medications						
Antilipemic agents	280,488 (53.5)	339,443 (35.5)	0.368	221,748 (49.3)	224,892 (50.0)	0.014
Glucocorticoids	229,533 (43.8)	233,132 (24.4)	0.418	172,776 (38.4)	173,723 (38.6)	0.004
Metformin	196,507 (37.5)	262,558 (27.5)	0.215	161,052 (35.8)	165,666 (36.8)	0.021
Insulins and analogues	162,095 (30.9)	153,145 (16.0)	0.357	118,941 (26.5)	117,951 (26.2)	0.005
COVID-19 vaccine	71,500 (13.7)	49,447 (5.2)	0.293	40,420 (9.0)	36,139 (8.0)	0.034
SGLT2 inhibitors	40,489 (7.7)	45,421 (4.8)	0.123	31,023 (6.9)	31,139 (6.9)	0.001
GLP-1 analogues	38,794 (7.4)	40,681 (4.3)	0.135	29,056 (6.5)	28,873 (6.4)	0.002
Laboratory data						
eGFR ≥60 mL/min/1.73 m^2^	356,811 (68.1)	451,451 (47.3)	0.432	290,659 (64.6)	295,126 (65.6)	0.021
Hemoglobin ≥12 g/dL	353,211 (67.4)	403,221 (42.2)	0.524	283,698 (63.1)	288,002 (64.0)	0.020
Albumin ≥3.5 g/dL	332,463 (63.5)	376,541 (39.4)	0.496	264,253 (58.8)	266,136 (59.2)	0.009
HbA1c ≥ 9%	66,123 (12.6)	81,743 (8.6)	0.132	52,319 (11.6)	52,786 (11.7)	0.003
Baseline healthcare utilization						
Critical care services	22,117 (4.2)	12,173 (1.3)	0.181	12,189 (2.7)	11,360 (2.5)	0.012
Hospitalization	201,066 (38.4)	212,699 (22.3)	0.356	150,131 (33.4)	148,820 (33.1)	0.006
Emergency department visit	185,458 (35.4)	185,380 (19.4)	0.364	137,389 (30.6)	138,167 (30.7)	0.004

Data are presented as numbers (%) or mean ± standard deviation. Propensity score matching was performed at a 1:1 ratio using a greedy nearest-neighbor algorithm with a caliper of 0.1 pooled standard deviations of the propensity-score logit. Standardized mean differences <0.10 were considered indicative of acceptable covariate balance. BMI, body mass index; CKD, chronic kidney disease; COVID-19, coronavirus disease 2019; eGFR, estimated glomerular filtration rate; GLP-1, glucagon-like peptide-1; HbA1c, hemoglobin A1c; SGLT2, sodium-glucose cotransporter 2; SMD, standardized mean difference; T2DM, type 2 diabetes mellitus.

### 3.2 Primary and secondary outcomes

After the 1-year landmark, patients with documented COVID-19 had a significantly higher incidence of overall dementia than matched controls (14,615 [3.25%] vs. 12,388 [2.75%]; HR 1.51, 95% CI 1.47–1.55, *p* < 0.001). The E-value analysis provided additional support for the robustness of the primary finding, with values of 2.39 for the point estimate and 2.30 for the lower confidence bound. These results suggest that residual confounding would need to be substantial to fully account for the observed association between documented COVID-19 and incident dementia. The Kaplan–Meier analysis showed a consistently lower dementia-free probability in the COVID-19 cohort than in matched controls through 6 years after the index date (log-rank *p* < 0.001; Figure 1).

**Figure 1:**
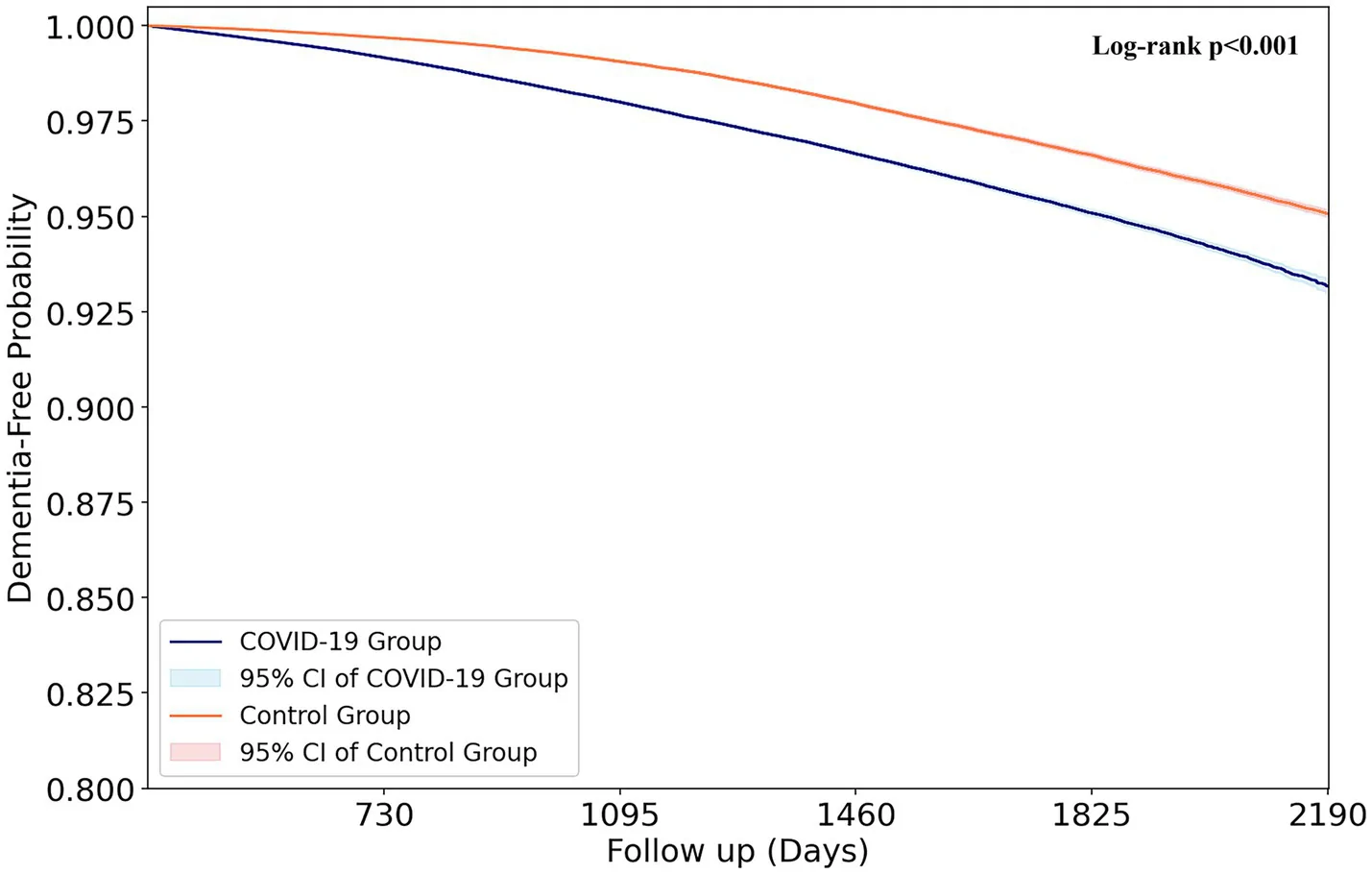
Dementia-free probability after the 1-year landmark period. Kaplan–Meier curves showing dementia-free probability among propensity score-matched older adults with type 2 diabetes with and without documented COVID-19. Follow-up began at the 1-year landmark point after the index date and continued through 2,190 days. The COVID-19 and control cohorts are represented by the blue and orange curves, respectively, with shaded areas indicating 95% confidence intervals. The lower dementia-free probability curve in the COVID-19 cohort indicates a greater risk of incident dementia during follow-up (log-rank *p* < 0.001).

Secondary outcomes were consistently elevated in the COVID-19 group: vascular dementia (HR 1.61, *p* < 0.001), Alzheimer’s disease (HR 1.41, *p* < 0.001), mild cognitive impairment (HR 1.66, *p* < 0.001), all-cause mortality (HR 1.56, *p* < 0.001), and cerebral infarction (HR 1.33, *p* < 0.001). The likelihood of having a recorded healthcare visit during follow-up was slightly lower in the COVID-19 group (HR 0.95, *p* < 0.001). Among negative-control outcomes, age-related cataract showed no significant excess risk (HR 0.97, *p* < 0.001), while benign skin neoplasm showed a modest elevation (HR 1.12, *p* < 0.001), both substantially smaller in magnitude than the dementia-related associations (Table 2). In the descriptive Aalen–Johansen analysis of the unmatched cohort, the cumulative incidence of dementia at the end of follow-up was higher in the COVID-19 group than in controls (6.77% vs. 4.62%), accompanied by a correspondingly greater cumulative incidence of competing mortality (14.84% vs. 9.12%) (Figure 2).

**Table 2:** One-year landmark analysis of incident dementia and related outcomes according to the documented COVID-19 status.

Outcome	COVID-19 group (*n* = 449,745)	Control group (*n* = 449,745)	HR (95% CI)	*p* value
	Events (%)	Events (%)		
Primary outcome				
Overall dementia	14,615 (3.25)	12,388 (2.75)	1.51 (1.47–1.55)	<0.001
Secondary outcomes				
Vascular dementia	2,610 (0.58)	2,120 (0.47)	1.61 (1.52–1.71)	<0.001
Alzheimer’s disease	3,402 (0.76)	3,174 (0.71)	1.41 (1.34–1.48)	<0.001
Mild cognitive impairment	5,698 (1.27)	4,511 (1.00)	1.66 (1.60–1.73)	<0.001
Mortality	36,723 (8.17)	29,226 (6.50)	1.56 (1.54–1.59)	<0.001
Cerebral infarction	27,804 (6.18)	24,621 (5.47)	1.33 (1.31–1.35)	<0.001
Follow-up healthcare encounter indicator				
Any healthcare visit	387,076 (86.07)	399,509 (88.83)	0.95 (0.95–0.96)	<0.001
Negative control outcomes				
Age-related cataract	38,570 (8.58)	44,614 (9.92)	0.97 (0.96–0.99)	<0.001
Benign skin neoplasm	6,061 (1.35)	6,411 (1.43)	1.12 (1.08–1.16)	<0.001

Data are presented as the number of events (%). Hazard ratios were estimated using Cox proportional hazards models in the propensity score–matched cohort. Follow-up began 365 days after the index date and continued until outcome occurrence, death, last available record, or 2,190 days after the index date, whichever occurred first. Hazard ratios >1 indicate higher risk in the COVID-19 group compared with the control group. CI, confidence interval; COVID-19, coronavirus disease 2019; HR, hazard ratio.

**Figure 2:**
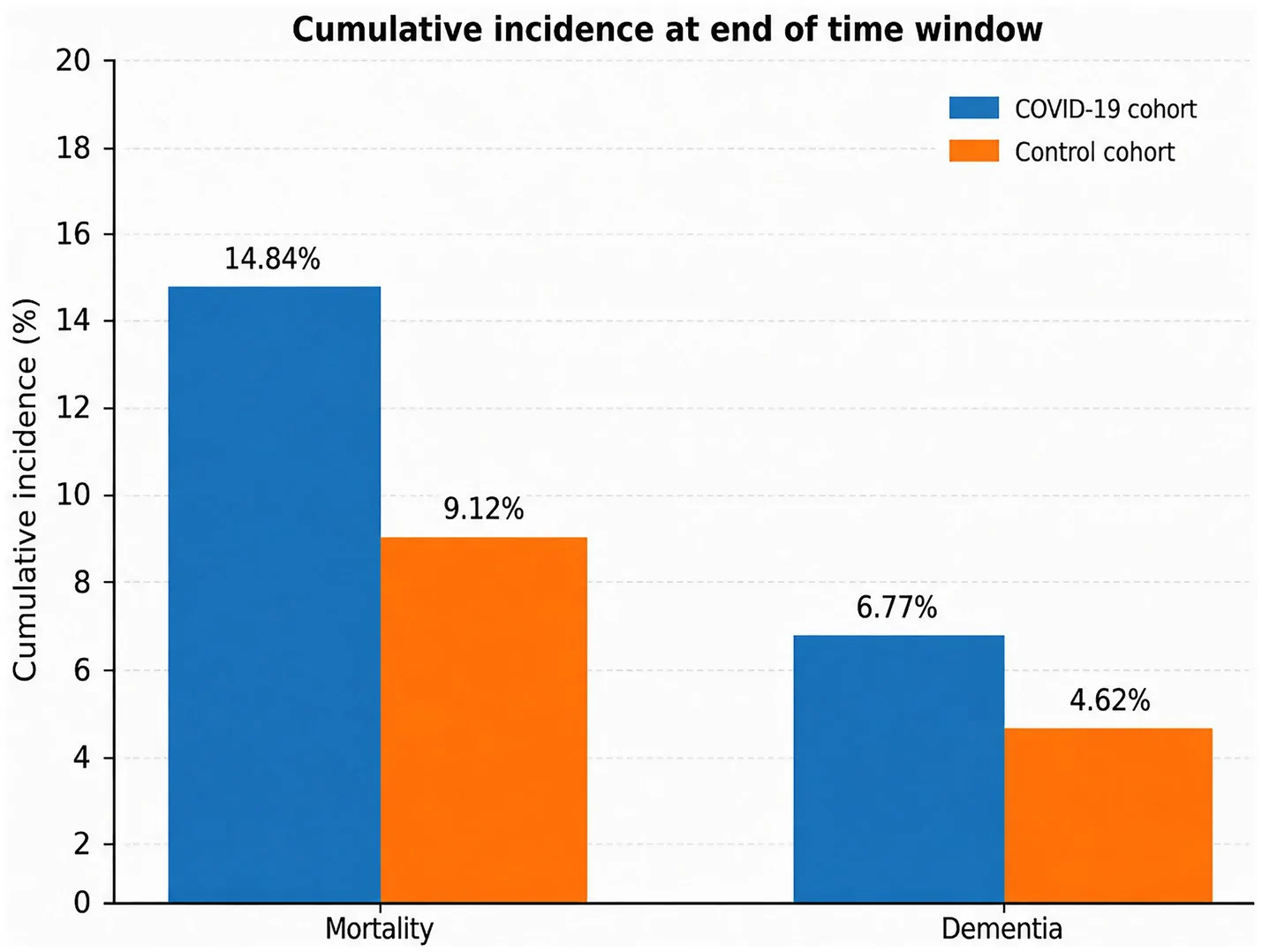
Descriptive competing-risk analysis for incident dementia with death treated as a competing event. Descriptive Aalen–Johansen competing-risk analysis showing the cumulative incidence of incident dementia among patients with type 2 diabetes with and without documented COVID-19 in the unmatched cohort, with death treated as a competing event. At the end of the observation window, the cumulative incidence of dementia was 6.77% in the COVID-19 cohort and 4.62% in the control cohort; the corresponding cumulative incidence of death was 14.84 and 9.12%, respectively. This analysis was descriptive and was not used for formal between-group statistical comparison.

### 3.3 Sensitivity analyses

The findings were consistent across three sensitivity models. Restricting to survivors throughout follow-up (Model I) yielded an HR of 1.52 for overall dementia (*p* < 0.001), and restricting to patients with at least one healthcare visit (Model II) yielded an HR of 1.53 (*p* < 0.001), both similar to the primary estimate. Excluding controls who subsequently developed documented COVID-19 (Model III) strengthened the association (HR 2.21, *p* < 0.001), consistent with attenuation of the primary estimate by exposure crossover. Similar patterns were observed for vascular dementia, Alzheimer’s disease, and mild cognitive impairment across all three models (Table 3). Across all three sensitivity models, the negative-control outcomes remained close to the null for age-related cataract or showed only modest elevations for benign skin neoplasm, with effect estimates substantially smaller than those observed for dementia-related outcomes. These findings further suggest that the observed associations were unlikely to be primarily driven by surveillance- or survival-related bias (Table 3).

**Table 3:** Sensitivity analyses for the association between documented COVID-19 and long-term risk of incident dementia.

Outcomes	Model I		Model II		Model III	
	HR (95% CI)	*p* value	HR (95% CI)	*p* value	HR (95% CI)	*p* value
Overall dementia	1.52 (1.48–1.57)	<0.001	1.53 (1.50–1.57)	<0.001	2.21 (2.14–2.27)	<0.001
Vascular dementia	1.64 (1.54–1.75)	<0.001	1.62 (1.52–1.71)	<0.001	2.51 (2.34–2.70)	<0.001
Alzheimer’s disease	1.45 (1.38–1.53)	<0.001	1.45 (1.38–1.52)	<0.001	1.84 (1.74–1.95)	<0.001
Mild cognitive impairment	1.68 (1.62–1.75)	<0.001	1.70 (1.63–1.77)	<0.001	2.11 (2.01–2.21)	<0.001
Mortality	NA	NA	1.60 (1.58–1.63)	<0.001	1.82 (1.79–1.85)	<0.001
Cerebral infarction	1.31 (1.29–1.33)	<0.001	1.33 (1.30–1.35)	<0.001	1.62 (1.59–1.65)	<0.001
Any healthcare visit	0.95 (0.94–0.95)	<0.001	0.94 (0.94–0.95)	<0.001	0.98 (0.98–0.99)	<0.001
Age-related cataract	0.96 (0.94–0.97)	<0.001	0.94 (0.93–0.95)	<0.001	0.99 (0.97–1.00)	0.074
Benign skin neoplasm	1.12 (1.09–1.17)	<0.001	1.10 (1.06–1.14)	<0.001	1.19 (1.15–1.24)	<0.001

Data are presented as hazard ratios with 95% confidence intervals and *p*-values. Model I was restricted to patients who survived during follow-up (COVID-19 group, *n* = 419,174; control group, *n* = 419,174). Model II was restricted to patients with at least one healthcare visit during follow-up (COVID-19 group, *n* = 391,307; control group, *n* = 391,307). Model III restricted the control group to patients without documented COVID-19 during follow-up to address potential exposure crossover (COVID-19 group, *n* = 413,260; control group, *n* = 413,260). Blank cells indicate outcomes that were not estimable or not applicable under the corresponding analytic restriction. CI, confidence interval; COVID-19, coronavirus disease 2019; HR, hazard ratio; NA, not applicable.

### 3.4 Landmark analyses

The hazard ratio for overall dementia increased progressively across sequential landmark periods: 1.51 (95% CI 1.47–1.55) at 1 year, 1.66 (95% CI 1.61–1.71) at 2 years, and 2.09 (95% CI 2.01–2.17) at 3 years (all *p* < 0.001) (Table 4). Vascular dementia, Alzheimer’s disease, and mild cognitive impairment showed comparable upward trends across landmarks. In contrast, the negative-control outcome of age-related cataracts remained close to or below the null throughout (HR 0.94–0.97, *p* < 0.001), while benign skin neoplasm showed only a modest, stable elevation (HR 1.12–1.23).

**Table 4:** Sequential landmark analyses of the association between documented COVID-19 and long-term risk of incident dementia.

Outcomes	1-year		2-year		3-year	
	HR (95% CI)	*p* value	HR (95% CI)	*p* value	HR (95% CI)	*p* value
Overall dementia	1.51 (1.47–1.55)	<0.001	1.66 (1.61–1.71)	<0.001	2.09 (2.01–2.17)	<0.001
Vascular dementia	1.61 (1.52–1.71)	<0.001	1.78 (1.66–1.91)	<0.001	2.40 (2.17–2.67)	<0.001
Alzheimer’s disease	1.41 (1.34–1.48)	<0.001	1.66 (1.57–1.76)	<0.001	2.15 (1.98–2.34)	<0.001
Mild cognitive impairment	1.66 (1.60–1.73)	<0.001	1.89 (1.81–1.98)	<0.001	2.63 (2.46–2.80)	<0.001
Mortality	1.56 (1.54–1.59)	<0.001	1.72 (1.69–1.76)	<0.001	1.97 (1.92–2.02)	<0.001
Cerebral infarction	1.33 (1.31–1.35)	<0.001	1.29 (1.27–1.32)	<0.001	1.32 (1.29–1.35)	<0.001
Any healthcare visit	0.95 (0.95–0.96)	<0.001	1.01 (1.01–1.02)	<0.001	1.03 (1.03–1.04)	<0.001
Age-related cataract	0.97 (0.96–0.99)	<0.001	0.94 (0.93–0.96)	<0.001	0.97 (0.95–0.99)	<0.001
Benign skin neoplasm	1.12 (1.08–1.16)	<0.001	1.15 (1.11–1.20)	<0.001	1.23 (1.17–1.29)	<0.001

Data are presented as hazard ratios with 95% confidence intervals and *p*-values. Landmark analyses were performed using 1-, 2-, and 3-year event-free periods after the index date. The 1-year landmark analysis corresponds to the primary analysis. Patients who developed the outcome of interest or died during each landmark period were excluded from the corresponding analysis. CI, confidence interval; COVID-19, coronavirus disease 2019; HR, hazard ratio.

### 3.5 Additional cohort analyses

In a separately matched cohort aged 50–65 years (*n* = 221,483 per group), documented COVID-19 was associated with an increased risk of overall dementia (755 [0.34%] vs. 574 [0.26%]; HR 1.63, 95% CI 1.46–1.82, *p* < 0.001), with concordant elevations in vascular dementia, Alzheimer’s disease, and mild cognitive impairment. Although the absolute incidence of dementia in this younger cohort was substantially lower than in the primary ≥65-year cohort (0.34% vs. 3.25% in the COVID-19 group), consistent with the lower baseline dementia risk in this age group, the relative hazard was of similar magnitude to the primary analysis (HR 1.63 vs. 1.51). This suggests that the association was not confined to the oldest patients (Table 5).

**Table 5:** Additional matched cohort analysis among adults aged 50–65 years with type 2 diabetes during the 2020–2022 index period.

Outcome	COVID-19 group (*n* = 221,483)	Control group (*n* = 221,483)	HR (95% CI)	*p* value
	Events (%)	Events (%)		
Overall dementia	755 (0.34)	574 (0.26)	1.63 (1.46–1.82)	<0.001
Vascular dementia	185 (0.08)	133 (0.06)	1.76 (1.40–2.21)	<0.001
Alzheimer’s disease	127 (0.06)	95 (0.04)	1.72 (1.31–2.25)	<0.001
Mild cognitive impairment	820 (0.37)	549 (0.25)	1.85 (1.66–2.07)	<0.001
Mortality	5,961 (2.69)	4,204 (1.90)	1.67 (1.61–1.74)	<0.001
Cerebral infarction	8,663 (3.91)	7,314 (3.30)	1.34 (1.30–1.38)	<0.001
Any healthcare visit	196,532 (88.74)	198,652 (89.69)	1.01 (1.01–1.02)	<0.001
Age-related cataract	14,605 (6.59)	15,495 (7.00)	1.06 (1.03–1.08)	<0.001
Benign skin neoplasm	2,797 (1.26)	2,581 (1.17)	1.24 (1.17–1.30)	<0.001

Data are presented as the number of events (%) unless otherwise indicated. This additional analysis used the same exposure definition, outcome definitions, covariate set, matching algorithm, and 1-year landmark design as the primary cohort but was restricted to adults aged 50–65 years. Hazard ratios were estimated using Cox proportional hazards models after propensity score matching. CI, confidence interval; COVID-19, coronavirus disease 2019; HR, hazard ratio.

In a cohort restricted to the Omicron-predominant period (July 2022–June 2023; *n* = 82,043 per group), the association with overall dementia persisted (2,180 [2.66%] vs. 1,709 [2.08%]; HR 1.49, 95% CI 1.40–1.59, *p* < 0.001), with an incidence and effect size closely comparable to the primary analysis (3.25% vs. 2.75%; HR 1.51), indicating that the association was not limited to earlier, more virulent pandemic phases. Negative-control outcomes in this variant-restricted cohort showed attenuated or non-significant differences (age-related cataract HR 0.96, *p* = 0.043; benign skin neoplasm HR 1.07, *p* = 0.194) (Table 6).

**Table 6:** Additional matched cohort analysis during the Omicron-predominant index period.

Outcome	COVID-19 group (*n* = 82,043)	Control group (*n* = 82,043)	HR (95% CI)	*p* value
	Events (%)	Events (%)		
Overall dementia	2,180 (2.66)	1,709 (2.08)	1.49 (1.40–1.59)	<0.001
Vascular dementia	381 (0.46)	269 (0.33)	1.66 (1.42–1.95)	<0.001
Alzheimer’s disease	461 (0.56)	410 (0.50)	1.32 (1.15–1.51)	<0.001
Mild cognitive impairment	815 (0.99)	660 (0.80)	1.45 (1.31–1.61)	<0.001
Mortality	4,901 (5.97)	3,630 (4.42)	1.56 (1.49–1.63)	<0.001
Cerebral infarction	3,963 (4.83)	3,843 (4.68)	1.16 (1.11–1.21)	<0.001
Any healthcare visit	68,612 (83.63)	73,073 (89.07)	0.90 (0.89–0.91)	<0.001
Age-related cataract	5,620 (6.85)	6,447 (7.86)	0.96 (0.93–1.00)	0.043
Benign skin neoplasm	716 (0.87)	764 (0.93)	1.07 (0.97–1.19)	0.194

Data are presented as the number of events (%) unless otherwise indicated. This additional analysis included patients indexed between 1 July 2022 and 30 June 2023, corresponding to the Omicron-predominant period. The same exposure definition, outcome definitions, covariate set, matching algorithm, and 1-year landmark design as the primary analysis were applied. Hazard ratios were estimated using Cox proportional hazards models after propensity score matching. CI, confidence interval; COVID-19, coronavirus disease 2019; HR, hazard ratio.

### 3.6 Multivariate and subgroup analyses

In the multivariate analysis, COVID-19 was independently associated with incident dementia (HR 1.67, 95% CI 1.64–1.71, *p* < 0.001) after adjustment for demographic and comorbidity covariates, alongside older age, cerebrovascular disease, nicotine dependence, and major depressive disorder as additional independent predictors (Table 7). In the subgroup analysis, the association between COVID-19 and dementia was significantly stronger among patients with poor glycemic control (HbA1c ≥ 9%: HR 1.66 vs. HbA1c < 9%: HR 1.47; p for interaction <0.001), chronic kidney disease (HR 1.63 vs. 1.48; *p* = 0.001), and dyslipidemia (HR 1.56 vs. 1.38; *p* < 0.001). Smaller but statistically significant effect modification was observed for sex, vaccination status, and hypertension, whereas no significant interaction was found for obesity, cerebrovascular disease, sleep disorders, or nicotine dependence (Table 8).

**Table 7:** Multivariable Cox regression analysis of factors associated with incident overall dementia.

Variables	HR (95% CI)	*p*-value
COVID-19 vs. control group	1.67 (1.64–1.71)	<0.001
Male	0.88 (0.86–0.90)	<0.001
Age at index	1.12 (1.11–1.12)	<0.001
White	0.92 (0.90–0.94)	<0.001
Hypertension	0.96 (0.93–0.98)	0.001
Dyslipidemia	0.79 (0.77–0.81)	<0.001
Neoplasms	0.82 (0.80–0.84)	<0.001
Overweight and obesity	0.87 (0.84–0.89)	<0.001
Ischemic heart diseases	1.06 (1.03–1.08)	<0.001
Sleep disorders	1.01 (0.98–1.03)	0.724
Chronic pain, not elsewhere classified	1.06 (1.03–1.09)	<0.001
Chronic kidney disease	1.04 (1.02–1.07)	0.002
Other anemias	1.12 (1.09–1.16)	<0.001
Heart failure	1.14 (1.11–1.18)	<0.001
Cerebrovascular diseases	1.54 (1.50–1.58)	<0.001
Nicotine dependence	1.36 (1.31–1.41)	<0.001
Major depressive disorder, recurrent	1.54 (1.46–1.63)	<0.001
Alcohol-related disorders	1.39 (1.29–1.50)	<0.001
Malnutrition	1.27 (1.17–1.39)	<0.001

CI, confidence interval; COVID-19, coronavirus disease 2019; HR, hazard ratio.

**Table 8:** Subgroup analyses of the association between documented COVID-19 and incident overall dementia.

Variables	HR (95% CI)	*p*-value	P for interaction
Male	1.57 (1.51–1.62)	<0.001	Reference
Female	1.46 (1.41–1.51)	<0.001	0.004
COVID-19 vaccine (+)	1.38 (1.29–1.47)	<0.001	Reference
COVID-19 vaccine (−)	1.51 (1.47–1.55)	<0.001	0.010
Hypertension (+)	1.53 (1.49–1.57)	<0.001	Reference
Hypertension (−)	1.42 (1.35–1.50)	<0.001	0.011
CKD (+)	1.63 (1.55–1.71)	<0.001	Reference
CKD (−)	1.48 (1.44–1.52)	<0.001	0.001
Obesity (+)	1.55 (1.48–1.62)	<0.001	Reference
Obesity (−)	1.54 (1.49–1.58)	<0.001	0.814
Dyslipidemia (+)	1.56 (1.51–1.60)	<0.001	Reference
Dyslipidemia (−)	1.38 (1.32–1.45)	<0.001	<0.001
Cerebrovascular disease (+)	1.51 (1.44–1.59)	<0.001	Reference
Cerebrovascular disease (−)	1.49 (1.45–1.54)	<0.001	0.654
Sleep disorder (+)	1.59 (1.51–1.67)	<0.001	Reference
Sleep disorder (−)	1.53 (1.48–1.57)	<0.001	0.200
Nicotine dependence (+)	1.50 (1.40–1.62)	<0.001	Reference
Nicotine dependence (−)	1.50 (1.46–1.54)	<0.001	0.998
HbA1c ≥ 9%	1.66 (1.56–1.77)	<0.001	Reference
HbA1c < 9%	1.47 (1.43–1.51)	<0.001	<0.001

CI, confidence interval; CKD, chronic kidney disease; COVID-19, coronavirus disease 2019; HbA1c, hemoglobin A1c; HR, hazard ratio.

## 4 Discussion

In this large propensity score-matched cohort study of older adults with type 2 diabetes, documented COVID-19 was associated with an increased long-term risk of incident dementia after a 1-year landmark period. The association was observed not only for overall dementia but also for vascular dementia, Alzheimer’s disease, mild cognitive impairment, cerebral infarction/stroke, and all-cause mortality. By extending follow-up to 6 years and incorporating sequential landmark analyses, negative-control outcomes, and a competing-risk framework, this study shows that the post-COVID dementia signal in this metabolically vulnerable population is not limited to the early post-infection period. Although residual confounding and detection-related bias cannot be completely eliminated, the consistency of findings across multiple analytic approaches supports a strong epidemiologic association between COVID-19 and delayed dementia risk among older adults with type 2 diabetes.

Although interest in the neurological sequelae of COVID-19 has grown substantially (Ahmad et al., 2022; Fiani et al., 2020; Shehata et al., 2021), evidence linking COVID-19 to incident dementia remains limited by relatively short follow-up and incomplete bias assessment (Zhang et al., 2025; Shan et al., 2024; Shrestha et al., 2024; Park et al., 2021). The majority of published cohort studies, including a large meta-analysis of 15 studies involving more than 26 million participants, have evaluated outcomes over only 6 to 24 months (Zhang et al., 2025). This short observation window makes it difficult to distinguish transient post-COVID diagnostic ascertainment from a potentially delayed neurodegenerative process. In addition, few studies have incorporated landmark analyses, negative-control outcomes, or competing-risk approaches, and these methods have rarely been used together to address surveillance bias, early outcome detection, and differential mortality.

In the current study, the sequential landmark analyses provide important insights into the temporal pattern of this association. If the observed increase in dementia risk were primarily attributable to short-term detection bias resulting from greater medical contact after COVID-19, the association would be expected to diminish as the interval between infection and outcome assessment lengthened. In contrast, the hazard ratio for overall dementia increased across progressively later landmark periods, from 1.51 at 1 year to 1.66 at 2 years and 2.09 at 3 years. Similar temporal patterns were observed for vascular dementia, Alzheimer’s disease, and mild cognitive impairment. These findings are inconsistent with a purely early ascertainment-driven explanation and may instead suggest a delayed or cumulative process after SARS-CoV-2 infection. Nevertheless, since landmark analyses cannot establish biological causality or fully eliminate residual bias, this pattern should be interpreted as supportive evidence for a persistent epidemiologic association rather than definitive proof of COVID-19-mediated neurodegeneration.

Negative-control, sensitivity, and competing-risk analyses provided supportive, although not definitive, evidence that the association was not primarily influenced by non-specific surveillance or survival-related bias. Age-related cataract, an outcome without a plausible mechanistic link to COVID-19 or dementia, showed no higher risk after infection and remained close to or below the null across landmark analyses. Benign skin neoplasm showed only modest elevations, with effect estimates substantially smaller than those observed for overall dementia and related cognitive outcomes. If the dementia association were mainly attributable to generalized healthcare-seeking or diagnostic intensity, similar increases would be expected across these unrelated outcomes. The observed discordance therefore suggests that differential surveillance is unlikely to fully explain the findings, although residual detection bias remains possible.

Sensitivity analyses further supported robustness. Restricting cohorts to patients who survived follow-up or had at least one recorded healthcare encounter yielded estimates similar to the primary analysis, arguing against differential mortality or healthcare retention as the dominant explanation. Excluding control patients who subsequently developed documented COVID-19 produced a stronger association, consistent with attenuation of the primary estimate by exposure crossover. The descriptive Aalen–Johansen analysis underscored substantial competing mortality in this older T2DM population. Together, these analyses show that the association persisted after addressing surveillance, survival, exposure misclassification, and competing mortality but should still be interpreted as supportive rather than causal evidence.

Subgroup and additional cohort analyses provided further context for interpreting the observed association. The association between COVID-19 and incident dementia was stronger among patients with poor glycemic control, chronic kidney disease, or dyslipidemia, suggesting that T2DM-related metabolic instability, endothelial dysfunction, and microvascular burden may amplify susceptibility to post-COVID neurological sequelae. These findings are biologically plausible because chronic hyperglycemia, endothelial dysfunction, and cerebrovascular injury are central mechanisms linking type 2 diabetes to cognitive decline (Chen et al., 2025; Luchsinger, 2012; Wang et al., 2014). Although female individuals had a higher overall risk of dementia in the multivariable model, the sex-stratified subgroup analysis addressed effect modification of the COVID-19–dementia association rather than baseline dementia susceptibility. COVID-19 was associated with increased dementia risk in both sexes, with a modestly stronger relative association in male individuals than in female individuals (HR 1.57 vs. 1.46; p for interaction = 0.004); however, this exploratory interaction should be interpreted cautiously. In addition, similar associations were observed in a separately matched cohort aged 50–65 years and in a cohort restricted to the Omicron-predominant period, suggesting that the findings were not confined to the oldest age group or to earlier pandemic phases. However, these subgroup and secondary cohort analyses should be interpreted as exploratory, and further studies are needed to clarify whether glycemic control, age, or viral variant period modifies the long-term neurocognitive risk after COVID-19.

Several biological mechanisms may plausibly link SARS-CoV-2 infection to delayed cognitive decline in patients with type 2 diabetes. Persistent systemic inflammation, neuroinflammatory activation, endothelial dysfunction, microvascular injury, thromboinflammatory responses, blood–brain barrier disruption, and amyloid-β dysregulation have all been implicated in post-COVID neurological sequelae (Ahmad et al., 2022; Greene et al., 2024; Dhariwal et al., 2024; Leng et al., 2023). These pathways overlap substantially with mechanisms involved in diabetes-related cognitive impairment, in which chronic hyperglycemia and insulin resistance promote sustained low-grade neuroinflammation, oxidative stress, endothelial dysfunction, microvascular injury, and impaired amyloid-β homeostasis (Chen et al., 2025; Yu et al., 2025; Kan et al., 2025; Feng and Gao, 2024; Duff et al., 2025). In patients with type 2 diabetes, pre-existing metabolic and cerebrovascular vulnerability may therefore lower the threshold for clinically apparent dementia after an additional inflammatory or vascular insult. Notably, recent longitudinal biomarker evidence showed that SARS-CoV-2 infection was associated with a reduced plasma Aβ42:Aβ40 ratio and, among more vulnerable older adults, lower Aβ42 and higher phosphorylated tau-181 levels (Duff et al., 2025). However, because this study was based on electronic health record data and did not include neuroimaging, biomarkers, longitudinal cognitive testing, or neuropathological information, these mechanisms could not be directly evaluated. Accordingly, the biological interpretation of our findings should be considered plausible but hypothesis-generating, rather than definitive evidence of COVID-19–mediated neurodegeneration.

Clinically, these findings suggest that older adults with type 2 diabetes who have had COVID-19 may warrant attention to cognitive health beyond the immediate post-infection period, particularly those with poor glycemic control or vascular risk factors. However, the modest absolute risk increase does not support routine dementia screening based solely on prior COVID-19. Instead, a history of COVID-19 may be considered as part of broader risk assessment when evaluating new cognitive symptoms or functional decline. Prospective studies with standardized cognitive testing are needed to clarify whether targeted long-term monitoring improves clinical outcomes.

Several limitations should be considered when interpreting these findings. First, dementia outcomes were identified using ICD-10-CM diagnostic codes, which are susceptible to misclassification and may underestimate subclinical or undiagnosed cognitive impairment. Moreover, no standardized cognitive test results were provided to validate coded diagnoses or evaluate longitudinal changes in cognitive performance. Second, the TriNetX platform does not provide detailed information about COVID-19 severity, treatment course, vaccination timing, the number and timing of reinfections, or virologically confirmed SARS-CoV-2 variants. Therefore, the potential association between repeated infections and dementia risk could not be reliably evaluated, and the Omicron-period analysis was based on calendar time rather than direct variant identification. Third, although death was addressed descriptively as a competing event using the Aalen–Johansen estimator, a formal competing-risk regression analysis with between-group statistical testing was not available within the platform. Fourth, residual confounding from unmeasured factors, such as education level, socioeconomic status, baseline cognitive function, frailty severity, lifestyle factors, differential access to care, age at T2DM onset, and diabetes duration, cannot be excluded. Although age at the index date was included in the matching model, the actual age at T2DM onset could not be reliably ascertained because the first recorded T2DM diagnosis in the electronic health record may not correspond to the true clinical onset of diabetes. In addition, because pioglitazone and acarbose (Tseng, 2018; Tseng, 2020) were used by only a very small proportion of patients, they were not included in the propensity score matching model; therefore, residual confounding related to these medications cannot be excluded. Fifth, the HbA1c subgroup analysis was based on available baseline measurements and could not account for changes in glycemic control during follow-up, and hypoglycemic events could not be comprehensively ascertained; therefore, these findings should not be interpreted as identifying an optimal longitudinal HbA1c target for dementia prevention or as accounting for the potential contribution of hypoglycemia to dementia risk. Finally, TriNetX includes patients from participating healthcare organizations rather than a population-based sampling frame. The aggregated analytic output did not permit organization- or country-specific analyses, and differences in coding practices, healthcare access, COVID-19 testing and management, and dementia ascertainment across sites may have influenced the observed estimates. Therefore, the generalizability of these findings to other healthcare systems or underrepresented populations should be interpreted with caution.

## 5 Conclusion

This large propensity score-matched cohort study found that documented COVID-19 was associated with an increased long-term risk of incident dementia among older adults with type 2 diabetes. The association persisted beyond the early post-infection period, increased across sequential landmark analyses, and remained robust in sensitivity analyses addressing survival, healthcare utilization, and exposure misclassification. Nevertheless, given the observational design, residual confounding and diagnostic misclassification remain possible. These results support a persistent epidemiologic association between COVID-19 and delayed dementia risk in a metabolically vulnerable population and highlight the need for prospective studies incorporating standardized cognitive assessments, neuroimaging, and biomarker data to clarify mechanisms and clinical implications.

## Data Availability

The datasets are not publicly available and are not available from the corresponding author because the study was conducted using the TriNetX Research Network, a federated electronic health record database. Patient-level data cannot be downloaded, exported, or shared by investigators due to data-use agreements, institutional restrictions, and privacy regulations. Only aggregate, de-identified results generated within the TriNetX platform were available to the investigators. The data underlying the analyses may be accessed only by authorized TriNetX users under appropriate institutional agreements. Requests to access the datasets should be directed to https://live.trinetx.com.
