# Supplementary material for: Long-term risk of incident dementia following documented COVID-19 among older adults with type 2 diabetes: a propensity score-matched cohort study
Source: Front Aging Neurosci. 2026 Sep 15;18:1927383. doi: 10.3389/fnagi.2026.1927383 (PMC13619926; doi:10.3389/fnagi.2026.1927383)
Supplement: Supplementary file 1 [file Supplementary_file_1.docx]

**Supplemental Table 1. Codes used to define exclusion criteria**

| Exclusion criterion | Code set / definition |
| --- | --- |
| Pre-existing or early dementia before or within 1 year after index date | ICD-10-CM F01, F02, F03, G30–G32 |
| Vascular dementia | ICD-10-CM F01 |
| Dementia in other diseases classified elsewhere | ICD-10-CM F02 |
| Unspecified dementia | ICD-10-CM F03 |
| Alzheimer’s disease and other degenerative diseases of the nervous system | ICD-10-CM G30–G32 |
| Early death within 1 year after index date | TriNetX deceased status |
| Ill-defined or unknown cause of mortality within 1 year after index date | ICD-10-CM R99 |
| Type 1 diabetes mellitus | ICD-10-CM E10 |
| Advanced chronic kidney disease, stage 4–5 | ICD-10-CM N18.4, N18.5 |
| End-stage renal disease | ICD-10-CM N18.6 |
| Dialysis dependence | ICD-10-CM Z99.2 |
| Intracranial injury | ICD-10-CM S06 |
| Multiple sclerosis | ICD-10-CM G35 |
| Epilepsy and recurrent seizures | ICD-10-CM G40 |
| Schizophrenia | ICD-10-CM F20 |
| Bipolar disorder | ICD-10-CM F31 |
| Parkinson’s disease | ICD-10-CM G20 |
| Prior use of donepezil | RxNorm 135447 |
| Prior use of rivastigmine | RxNorm 183379 |
| Prior use of galantamine | RxNorm 4637 |
| Prior use of memantine | RxNorm 6719 |
| Conductive and sensorineural hearing loss | ICD-10-CM H90 |
| Other and unspecified hearing loss | ICD-10-CM H91 |
| Documented COVID-19 in the control cohort | ICD-10-CM U07.1 |
| Positive SARS-CoV-2 RNA test in the control cohort | TriNetX laboratory term TNX:9088, SARS coronavirus 2 and related RNA [Presence] |

**Supplemental Table 2. Codes for variables used in propensity score matching**

| Matching variable | Code set / definition |
| --- | --- |
| Age at index | TriNetX demographic variable |
| Sex | TriNetX demographic variable |
| White race | TriNetX race variable |
| Black or African American race | TriNetX race variable |
| Asian race | TriNetX race variable |
| Body mass index ≥30 kg/m² | TriNetX BMI laboratory/vital record ≥30 kg/m² |
| Essential hypertension | ICD-10-CM I10 |
| Dyslipidemia / disorders of lipoprotein metabolism and other lipidemias | ICD-10-CM E78 |
| Neoplasms | ICD-10-CM C00–D49 |
| Overweight and obesity | ICD-10-CM E66 |
| Ischemic heart diseases | ICD-10-CM I20–I25 |
| Sleep disorders | ICD-10-CM G47 |
| Chronic pain, not elsewhere classified | ICD-10-CM G89.2 |
| Vitamin D deficiency | ICD-10-CM E55 |
| Chronic kidney disease | ICD-10-CM N18 |
| Anxiety disorders | ICD-10-CM F41 |
| Type 2 diabetes mellitus with kidney complications | ICD-10-CM E11.2 |
| Other anemias | ICD-10-CM D64 |
| Heart failure | ICD-10-CM I50 |
| Type 2 diabetes mellitus with neurological complications | ICD-10-CM E11.4 |
| Cerebrovascular diseases | ICD-10-CM I60–I69 |
| Diseases of liver | ICD-10-CM K70–K77 |
| Other chronic obstructive pulmonary disease | ICD-10-CM J44 |
| Nicotine dependence | ICD-10-CM F17 |
| Type 2 diabetes mellitus with circulatory complications | ICD-10-CM E11.5 |
| Type 2 diabetes mellitus with ophthalmic complications | ICD-10-CM E11.3 |
| Sepsis | ICD-10-CM A41 |
| Major depressive disorder, recurrent | ICD-10-CM F33 |
| Alcohol-related disorders | ICD-10-CM F10 |
| Problems related to care-provider dependency | ICD-10-CM Z74 |
| Repeated falls | ICD-10-CM R29.6 |
| Malnutrition | ICD-10-CM E40–E46 |
| Antilipemic agents | TriNetX therapeutic class CV350 / ATC C10 |
| Insulins and analogues | ATC A10A |
| Metformin | RxNorm 6809 |
| Glucocorticoids | ATC H02AB |
| Sodium-glucose cotransporter 2 inhibitors | ATC A10BK |
| Glucagon-like peptide-1 receptor agonists / analogues | ATC A10BJ |
| COVID-19 vaccination | TriNetX vaccine term 213 |
| Estimated glomerular filtration rate ≥60 mL/min/1.73 m² | TriNetX laboratory term for eGFR; threshold ≥60 mL/min/1.73 m² |
| Hemoglobin ≥12 g/dL | TriNetX laboratory term for hemoglobin; threshold ≥12 g/dL |
| Albumin ≥3.5 g/dL | TriNetX laboratory term for albumin; threshold ≥3.5 g/dL |
| Hemoglobin A1c ≥9% | TriNetX laboratory term TNX:9037; HbA1c ≥9% |
| Critical care services | TriNetX procedure term 1013729, Critical Care Services |
| Emergency department visit | TriNetX visit term EMER, Visit: Emergency |
| Hospitalization | TriNetX visit term IMP, Visit: Inpatient Encounter |
